# Effects of Dietary Tussah Immunoreactive Pupa Powder on Growth, Gonad Quality, Antioxidant Capacity, and Gut Microbiota of the Sea Urchin *Strongylocentrotus intermedius*

**DOI:** 10.3390/biology14070874

**Published:** 2025-07-17

**Authors:** Shufeng Li, Fenglin Tian, Weiyan Li, Haoran Xiao, Ye Tian, Yajie Deng, Lingshu Han, Chong Zhao, Jun Ding

**Affiliations:** 1 Liaoning Provincial Key Laboratory of Northern Aquatic Germplasm Resources and Genetics and Breeding, Dalian Ocean University, Dalian 116023, China; 2 Key Laboratory of Mariculture & Stock Enhancement in North China’s Sea, Ministry of Agriculture and Rural Affairs, Dalian Ocean University, Dalian 116023, China; 3 Dalian Jinshiwan Laboratory, Dalian 116034, China

**Keywords:** *Strongylocentrotus intermedius*, IPP, antimicrobial peptides, gonad quality, gut microbiota

## Abstract

**Simple Summary:**

Tussah immunoreactive pupa powder (IPP) is a novel aquafeed additive used in aquaculture, composed of various active substances. Therefore, we incorporated IPP into the feed and conducted a 60-day experiment. The results indicated that IPP had a positive effect on the growth, gonad quality, antioxidant capacity, and gut health of sea urchins. This study provides theoretical insights into the healthful farming of sea urchins and further extends the application of IPP in aquaculture.

**Abstract:**

Tussah immunoreactive pupa powder (IPP) is composed of various active substances. We speculated that it has the potential to improve key economic traits of sea urchins. Therefore, we conducted a 60-day experiment to examine the effects of IPP on growth, antioxidant capacity, gonad quality, and gut microbiota of sea urchins (*Strongylocentrotus intermedius*). The experiment involved the preparation of a kelp group and four types of feed containing 0% (the control group), 0.5%, 1.0%, and 1.5% IPP. The results indicated that IPP had no significant impact on the survival of sea urchins (*p* > 0.05). Firstly, adding IPP promoted the growth of sea urchins. The 1.0% IPP group showed the highest weight gain rate among the feed group, significantly higher than that of the control group (*p* < 0.05). Secondly, compared with the kelp group, the addition of IPP significantly improved the growth and quality of sea urchin gonads (*p* < 0.05), which demonstrated certain industrial value. Thirdly, following the addition of IPP, the activities of SOD, CAT, and POD significantly increased in comparison to the control group (*p* < 0.05). Lastly, added IPP increased the abundance of *Firmicutes*, *Bacteroidetes*, and *Rhodobacteraceae*, while reducing the abundance of *Ralstonia* and *Vibrio*. This indicates that added IPP may improve the digestive function and gut health of sea urchins. Overall, added IPP can improve certain economic traits and antioxidant capacity of sea urchins. This manuscript provides a theoretical reference for the healthful aquaculture of *S. intermedius*.

## 1. Introduction

The gonads of sea urchins are not only concerned for their delectable flavor but are acknowledged for their nutritional richness. They are recognized internationally as a rare culinary ingredient [1,2]. Sea urchin (*Strongylocentrotus intermedius*) is an important economic aquaculture species in northern China. It is native to the Hokkaido region of Japan and was introduced to China in 1989 by Dalian Ocean University [3]. Sea urchins primarily feed on macroalgae, such as *Laminaria japonica* and *Undaria pinnatifida* [4,5,6]. However, the supply of macroalgae is seasonal, and using algae as the primary aquaculture feed may lead to issues such as nutritional unitary and low feed conversion rates in sea urchins [7,8,9]. At the same time, global stocks of sea urchins have been overfished due to their status as a high-quality product, valued for their nutritional value, unique biochemical composition, and acquired taste [10]. Consequently, commercial farming (e.g., sea-based rearing systems) seems to be a suitable approach for sea urchin aquaculture [10,11]. It is necessary to utilize formulated diets to address the insufficient supply of macroalgae during sea urchin farming [5,10,11]. However, sea urchins fed with formulated diets present several disadvantages that require solutions, including low weight gain rates, poor gonad color, and increased abundance of *Vibrio* in the intestine [12,13,14].

Tussah immunoreactive pupa powder (IPP) is a novel aquafeed additive utilized within aquaculture [15,16]. IPP contains many active substances obtained from the Tussah (*Antheraea pernyi*) through the artificial injection of probiotics. This process induces the *A. pernyi* pupa to produce various active polypeptides and proteins, including antimicrobial peptides and lysozyme [17]. Since the discovery of the first antimicrobial peptide, it has garnered widespread concern among researchers due to its remarkable antimicrobial activity against pathogens [18,19,20,21,22]. Antimicrobial peptides have already been applied in aquaculture due to their various benefits, such as stability, water solubility, and safety for humans [23,24]. During the aquaculture period, the use of antimicrobial peptides can positively impact the growth rate and immune response of organisms [25,26], which can also improve the survival rate of fish in the aquaculture process [27]. Therefore, it is regarded as a new generation of green feed additives. Studies have shown that using feed containing IPP with antimicrobial peptides in the feeding process of sea cucumbers (*Apostichopus japonicus*) can significantly improve their weight gain rate, disease resistance, and positively influence gut microbiota regulation [15]. Currently, the application of IPP in sea urchin feed is limited. Given the excellent properties of IPP, we speculate that it has the potential to improve the adverse effects (e.g., low weight gain rates, poor gonad color, and increased abundance of *Vibrio* in the intestine) caused by the formulated diets.

In this study, we reasonably added IPP into the feed with the aim of investigating its impact on key economic traits (e.g., growth and gonad quality), antioxidant capacity, and gut microbiota of sea urchins. Ultimately, we reference the experiment results to determine the optimal addition ratio of IPP. This study provides theoretical insights into the healthy farming of sea urchins and further extends the application of IPP in aquaculture.

## 2. Materials and Methods

### 2.1. Sea Urchins

The feeding experiments lasted for 60 days, and they were conducted at the Key Laboratory of Mariculture and Stock Enhancement in North China’s Sea, Ministry of Agriculture and Rural Affairs, Dalian Ocean University, on 15 March 2024. All sea urchins used in the experiments were obtained from local farms in Dalian. We acclimated sea urchins for 14 days before the feeding experiments. To maintain water quality and hydrology stability, we removed residual feed and feces after each feeding. The water was regularly changed with fresh seawater, replacing one-third of the total water volume each time. During the farming period, we used a water quality measurement instrument (YSI ProQuatro, Snellville, GA, USA) to monitor hydrological variations. The water temperature was maintained throughout the experiment at 13–15 °C, and the salinity was at 32 ± 0.5‰. We used an oxygen generator (HAILEA, Guangzhou, China) to supply oxygen to the aquaculture water, maintaining the dissolved oxygen level at 7.0 ± 0.5 mg/L.

### 2.2. Experimental Design

Before starting the feeding experiment, 125 similar-sized and healthy sea urchins (test diameter 40.89 ± 1.73 mm; body weight 17.27 ± 0.51 g) were selected and randomly divided into 5 groups. The same group of sea urchins was randomly and evenly distributed among five cylindrical net cages (each about 25 cm in diameter and 40 cm in height). A petri dish was placed at the bottom of each cage to prevent feed from falling through the mesh gaps. All cages were placed in a 500 L tank. The experiment was conducted in five groups: a kelp group (which also served as the industrial control group), a 0% IPP group (control group), a 0.5% IPP group, a 1.0% IPP group, and a 1.5% IPP group. Each cage was fed kelp or feed twice daily during the experimental period, feeding about 5% of the sea urchin’s weight at one time.

### 2.3. Experimental Feed Preparation

The experimental feeds were made with fish meal, soybean meal, and wheat flour as the main ingredients. The main protein sources were soybean meal and fish meal, while wheat flour was the primary carbohydrate source. IPP was added to the basal feed at concentrations of 0%, 0.5%, 1.0%, and 1.5%, resulting in four experimental feeds. Before feed preparation, all solid ingredients were ground and passed through an 80 μm sieve. After precise weighing, the solid components were thoroughly mixed and combined with the appropriate amount of oil according to each feed formula. Approximately 30% water was added to ensure thorough mixing. Finally, the mixture was pelletized into feed pellets measuring 2 mm × 20 mm by using a pelletizer (DES-TS1280, Jinan, China) and dried at 50 °C for about 24 h. The dried pellets were packed in sealed bags and stored at −20 °C until use. The nutritional composition of the basal feed is shown in Table S1.

### 2.4. Sample Collection

After the 60-day feeding experiment, the sea urchins were fasted for 24 h. Sea urchins in each group were subsequently counted and measured for growth traits. The samples (e.g., coelomic fluid, gonads, and intestines) were collected from three sea urchins per group randomly, which combined to create a single composite sample. The collected body cavity fluid and intestines were separately pooled to form one biological replicate [14]. This process was carried out three times, resulting in three pooled replicates for each group. Three mixed coelomic fluid samples were rapidly frozen in liquid nitrogen for subsequent analysis of antioxidant capacity. The gonads from each sea urchin were divided into five parts: the first gonad was used for texture properties analysis, the second gonad was temporarily preserved on ice and utilized for color evaluation, and the third and fourth gonads were combined for subsequent analysis of nutrient profiles. Finally, three mixed intestinal samples were collected from each group for analysis of the gut microbiota. After all the samples were dissected, we stored them at −80 °C.

### 2.5. Growth Indicators

The body weight (g), test diameter (mm), and test height (mm) of each experimental group of sea urchins were measured and recorded by using an electronic balance or a vernier caliper (*n* = 3):

(1) $$WGR\%=\left(m_{t}-m_{o}\right)/m_{0}\times 100$$

(2) $$GSI\%=\left(m_{g}/m_{t}\right)\times 100$$

(3) $$SR\%=\left(N_{0}/N_{t}\right)\times 100$$

In the above formula, *m*_0_ denotes the initial body weight (g), *m_t_* denotes the final body weight (g), *m_g_* denotes the total gonad wet weight per sea urchin (g), *N*_0_ denotes the initial number of sea urchins, and *N_t_* represents the final number of sea urchins.

### 2.6. Antioxidant Capacity

The associated processes were referenced by Li et al. [5]. The activities of catalase (CAT), peroxidase (POD), and superoxide dismutase (SOD) were quantified utilizing assay kits obtained from the Nanjing Jiancheng Bioengineering Institute. The total protein content was determined utilizing Bradford’s method [28], with bovine serum albumin serving as the standard for quantification criteria. All experimental procedures and formulations were conducted in strict accordance with the manufacturer’s instructions.

### 2.7. Gonad Color Determination

We randomly sampled three sea urchin gonad specimens from each experimental group, placed them within transparent, sealed bags, and pulverized them to achieve a uniform texture devoid of air bubbles. The color was measured using a colorimeter (PANTONE, Carlstadt, NJ, USA) to determine the values of lightness (L*), redness (a*), and yellowness (b*). We recorded the value as Δ*E* afterwards. A lower Δ*E* value means a closer match to the standard color. The standard colors were light-orange–yellow (L* = 68.9, a* = 28.7, b* = 60.4) and light yellow (L* = 74.6, a* = 28.7, b* = 66.1), as referenced by McBride et al. [29]:

Δa* = a* − 28.7 (4)

Δb* = b* − 60.4 (or 66.1) (5)

Δ*E* = SQRT (ΔL* 2 + Δa* 2 + Δb* 2) (6)

In the formula, Δ*E* is the difference between the gonad color and the standard color for each sea urchin, a* is redness, and b* is yellowness.

### 2.8. Gonad Texture Properties

We randomly selected three gonad samples from each sea urchin experimental group for texture properties’ analysis. We performed measurements three times for each sample. The texture of sea urchin gonads was referenced by Martinez et al. [30] and determined using a texture analyzer (TMS-Pro, FTC, Great Neck, NY, USA) [29]. We used a 20 mm-diameter cylindrical probe to compress each gonad sample twice at a speed of 60 mm/min to half of its original height. The definitions of hardness, adhesiveness, springiness, cohesiveness, gumminess, and chewiness (*mJ*) can be seen in Table S2.

### 2.9. Routine Nutritional Composition

The proximate composition analysis of sea urchin gonads and feed was conducted in accordance with the Association of Official Analytical Chemists (AOAC) methods [14,31]. We randomly selected three gonad samples from each experimental group for analysis (*n* = 3). The moisture content was determined using the wet weight method. We placed the gonads in an oven at 105 °C and dried them until their weight was constant, then calculated the difference between the weight before and after drying. Crude protein content was determined using the Kjeldahl method [32]. Crude fat was determined using the Soxhlet extraction method [33].

### 2.10. Sequencing of Gut Microbiota

The specific analysis process was referenced by Li et al. [13]. In simple terms, total DNA was extracted using the OMEGA E.Z.N.A™ Mag-Bind Soil DNA Kit (OMEGA Bio-Tek, Norcross, GA, USA). We utilized a NanoDrop 2000 spectrophotometer (Thermo Fisher Scientific Inc., Waltham, MA, USA) to measure the concentration and purity of the DNA. After that, the FR-1000 device was utilized to assess the integrity of the DNA. The V3–V4 region of the bacterial 16S RNA gene was amplified utilizing 341F 5′-CCTACGGGNGGCWGCAG-3′ and 805R 5′-GACTACHVGGGTATCTAATCC-3′ [34]. Then, purified amplicons were obtained, utilizing the Illumina MiSeq 2 × 300 bp platform (Illumina, San Diego, CA, USA) for high-throughput sequencing [35]. We utilized USEARCH (v.11.0.667) to conduct OUT clustering with a 97% similarity threshold and employed it to eliminate chimeric sequences. The alpha diversity analysis (e.g., Chao1, abundance-based coverage estimator, Simpson index, and Shannon diversity) was conducted utilizing the summary single command of MOTHUR software (v.1.43.0) (http://www.mothur.org, accessed on 14 July 2025). Chao1 values and ACE values were based on the measured OTU number of the samples. Subsequently, alpha diversity analysis was conducted on the Sangon Biotech Cloud (https://ngs.sangon.com/, accessed on 14 July 2025) (Sangon Biotech, Shanghai, China).

### 2.11. Data Analysis

We utilized SPSS 22.0 software (IBM Corp., Armonk, NY, USA) for data analysis. The Shapiro–Wilk and Levene’s tests were conducted to analyze the normality of the data and the homogeneity of variances. The ANOVA of variance and Duncan’s multiple comparison test were used to analyze the differences in the measured indicators among the treatment groups, with *p* < 0.05 considered statistically significant [34]. The statistical results were expressed as mean ± standard deviation (Mean ± S.D).

## 3. Results

### 3.1. Sea Urchins and Gonads Growth

As shown in Table 1, the survival rate of sea urchins was 100% in all experimental groups. The weight gain rate of sea urchins in the group with added IPP was significantly higher than that of the control group (*p* < 0.05). Among the feed groups, the 1.0% IPP group had the highest body weight gain rate of 77.33%. Although the 1.0% IPP group had the highest body weight gain rate in all feed groups, it was slightly lower than that of the kelp group.

Based on the analysis of sea urchin gonad weight data (Table 1), the wet gonad weight of sea urchins in the IPP feed groups was slightly higher than that of the kelp group. Among them, the 1.0% IPP group had the highest gonad weight, which was significantly higher than that of the kelp and control groups (*p* < 0.05). However, there was no significant difference compared to the 1.5% IPP group (*p* > 0.05). As the content of IPP in the feed rose, the wet weight of the gonads initially increased and subsequently decreased, peaking in the 1.0% IPP group.

### 3.2. Antioxidant Enzyme Activity

**Figure 1:**
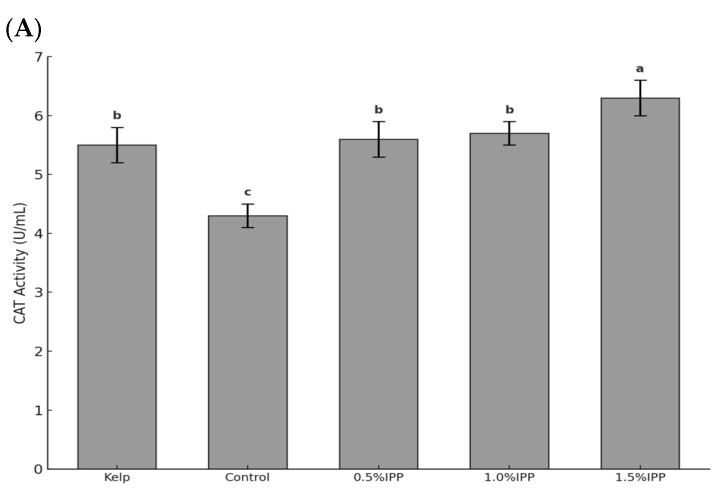

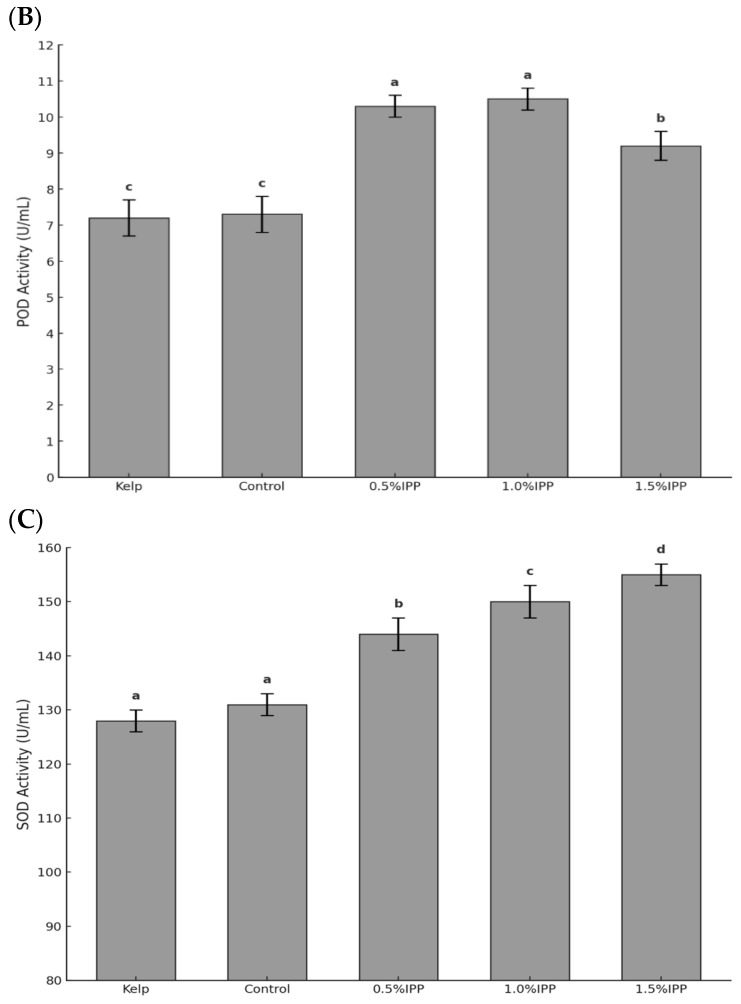
Effect of adding IPP to the feed on sea urchin antioxidant enzyme activity. Note: (**A**) CAT activity of sea urchins in each experimental group after 60 days. (**B**) POD activity of sea urchins in each experimental group after 60 days. (**C**) SOD activity of sea urchins in each experimental group after 60 days. Different letters indicate significant differences between groups (*p* < 0.05), while the same letters indicate no significant differences between groups (*p* > 0.05).

As shown in Figure 1, after the addition of IPP, the activities of POD and SOD were significantly higher than those in the kelp and control groups (*p* < 0.05). The highest activities of CAT and SOD were found in the 1.5% IPP group, which were significantly higher than those in the other groups (*p* < 0.05). Additionally, the activities of CAT and SOD in the feed group increased with the addition of IPP, peaking in the 1.5% IPP group. The highest POD activity was found in the 1.0% IPP group, which was significantly higher than that of the kelp and control groups (*p* < 0.05).

### 3.3. Gonad Color

As shown in Table 2, the values of Δ*E*_1_ and Δ*E*_2_ for the feed group exhibited a tendency to decrease with the addition of IPP. The gonad lightness was highest in the 1.5% IPP group and significantly higher than that of the kelp group *(p* < 0.05). The redness and yellowness values were highest in the kelp group, which were also significantly higher than those of the other experimental groups (*p* < 0.05).

### 3.4. Gonad Texture

As shown in Table 3, the gonad hardness, cohesiveness, gumminess, and chewiness of the 1.0% IPP group were significantly higher than those of the kelp and control groups (*p* < 0.05). Additionally, the hardness of sea urchin gonads showed an initial rise followed by a decline as IPP increased, peaking in the 1.0% IPP group. However, there was no significant difference in the adhesiveness and springiness of sea urchin gonads among the groups (*p* > 0.05).

### 3.5. Routine Nutritional Composition of Gonads

As shown in Table 4, the gonad moisture was lowest in the 1.0% IPP group, which was also significantly lower than that of the control and kelp groups (*p* < 0.05). Concurrently, the crude fat content reached its highest level in the 1.0% IPP group and was significantly higher than that in the other feed groups (*p* < 0.05). However, it remained lower than that in the kelp group, with no significant difference (*p* > 0.05). The crude protein content in the gonads showed an upward trend with the increasing addition of IPP, peaking in the 1.5% IPP group, which was significantly higher than that in the other groups (*p* < 0.05).

### 3.6. Gut Microbiota

This study clustered 3195 operational taxonomic units (OTUs) through 16S rRNA sequencing. According to the results of the rarefaction curves, the sequencing depth of all samples was sufficient to support further analysis. The ACE and Chao1 indices indicated that the abundance of gut microbiota decreased with IPP addition and peaked in the 0.5% IPP group. Additionally, the results of the Shannon and Simpson indices indicated that added IPP promoted an increase in gut microbial diversity, reaching a peak in the 1.0% IPP group. Furthermore, gut microbial diversity in all added IPP groups was higher than that in the kelp and control groups (*p* < 0.05; Table 5).

**Figure 2:**
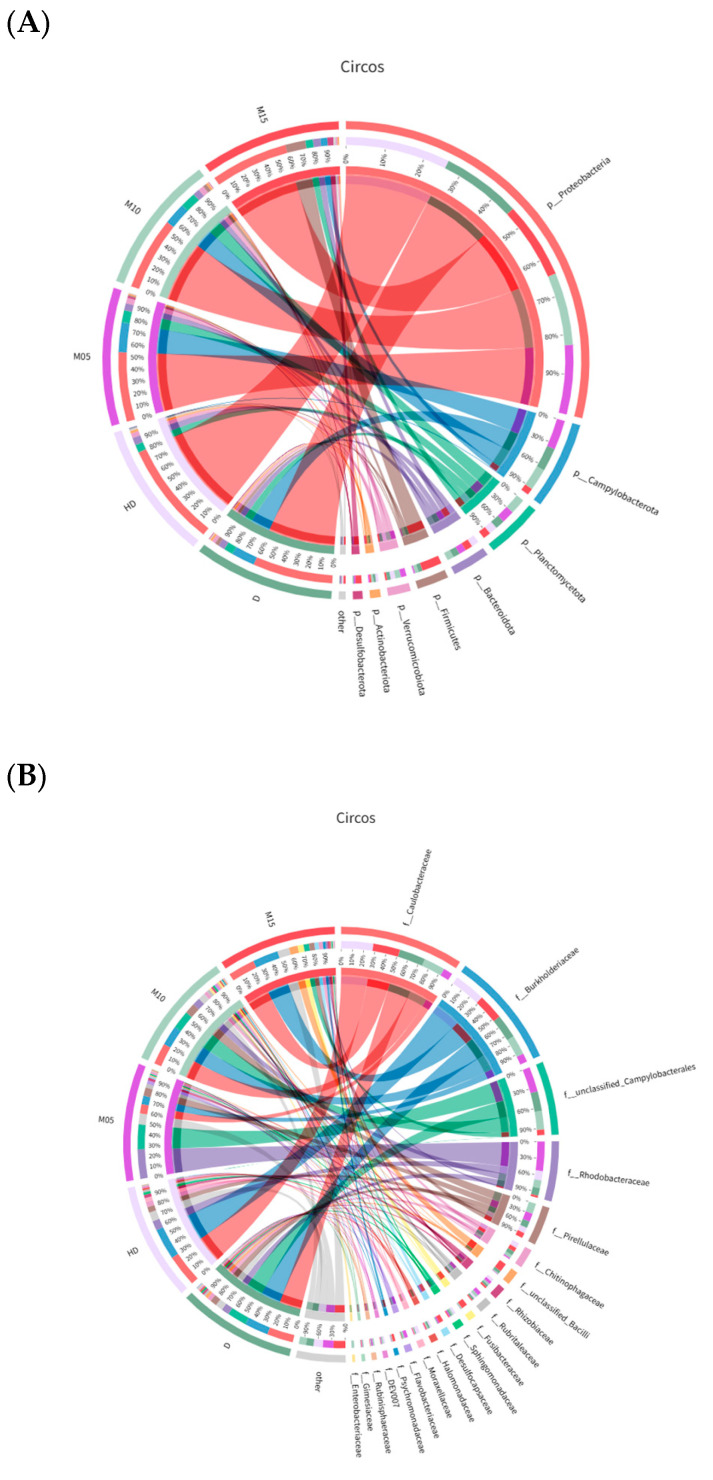

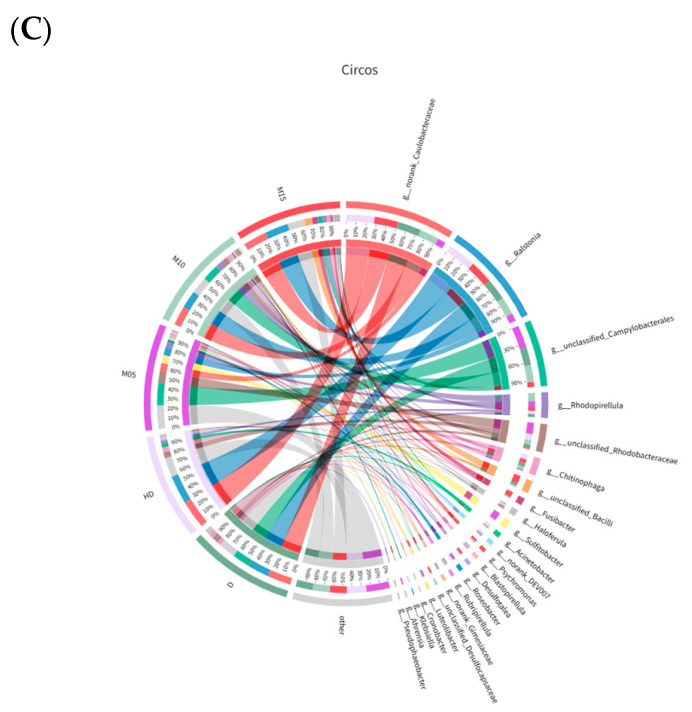
Taxonomic classification of reading at the phylum (**A**), family (**B**), and genus (**C**) taxonomic levels.

In this study, *Proteobacteria*, *Campylobacterota*, and *Planctomycetota* were observed to dominate the bacterial composition across all groups. Following the addition of IPP, the abundance of *Firmicutes* and *Bacteroidetes* increased in the intestine of the sea urchin. Concurrently, the relative abundance of Proteobacteria also experienced a rise, reaching 3.8%, 7.0%, and 7.4% in the groups receiving 0.5%, 1.0%, and 1.5% IPP, respectively. At the family level, *Caulobacteraceae* and *Burkholderiaceae* were the two most prevalent bacterial families across all groups. Furthermore, the abundance of *Rhodobacteraceae* showed an upward trend as the concentration of IPP increased, attaining a maximum of 13.7% in the 0.5% IPP group. At the same time, the addition of IPP was found to decrease the abundance of *Vibrio* in the increase of sea urchins. At the genus level, *Caulobacteraceae*, *Ralstonia*, and *Campylobacterales* were the dominant entities in the bacterial composition. The abundance of *Ralstonia* showed an upward trend with the increased addition of IPP, peaking in the 1.5% IPP group and at an abundance of 8.9% (Figure 2).

## 4. Discussion

### 4.1. Effects of Adding IPP to the Feed on Growth of Sea Urchins

In order to satisfy the nutritional requirements of sea urchins during aquaculture and reduce production costs, previous researchers have conducted various studies concerning feeds [5,6,13]. In this study, we added IPP into the sea urchin feed and observed that it can promote their growth. Among all feed groups, the 1.0% IPP group showed the highest weight gain rate, which was significantly greater than that of the control group (*p* < 0.05). IPP comprises various active substances [17], with its protein content capable of reaching 67% to 70% [36]. The importance of protein in aquaculture is self-evident, and adequate protein intake is important for the growth of aquatic organisms [37]. Previous studies have demonstrated that the addition of IPP to the feed can enhance the growth of *A. japonicus* [15] and *Takifugu rubripes* [38]. This may be related to the increase in protein absorption. Meanwhile, the 1.0% IPP group had the highest weight gain rate among all feed groups. Therefore, we speculated that adding IPP can increase the crude protein levels and individual protein absorption, consequently improving the growth of sea urchins.

However, as the amount of IPP increased, the sea urchin weight gain rate initially rose, followed by a decline. The addition of 1.0% IPP demonstrated the most pronounced effect on sea urchin growth. The study by Ma et al. showed that adding 1.5% IPP to the feed reduced the weight gain rate of Yesso scallops (*Patinopecten yessoensis*) compared to the 1.0% IPP group [16]. This finding is consistent with our results. Based on their conclusion, we reasonably inferred the similarity of findings in our study. We speculate that the addition of high levels of IPP may be linked to toxicity or disruptions in the gut health of sea urchins, subsequently influencing their growth. However, the underlying mechanism by which IPP influences their growth is unknown and requires further study. Overall, the appropriate addition of IPP had a certain positive effect on the economic traits of sea urchins.

### 4.2. Effects of Adding IPP to the Feed on Gonad Traits of Sea Urchins

#### 4.2.1. Effects of Adding IPP to the Feed on Gonad Growth of Sea Urchins

As the sole edible ingredient of the sea urchin, the gonads are not only rich in nutrients and active substances but are exceptionally palatable [1,2]. In this study, the 1.0% IPP group exhibited the highest wet gonad weight, which was significantly higher than that of the kelp group (*p* < 0.05). IPP contains a higher protein level than soybean meal and other feed ingredients [36]. Furthermore, it possesses a higher concentration of globulins and albumin, which are absorbed more easily than other protein sources [39]. Adequate protein intake significantly increases the gonad index in sea urchins [40]. The increased gonad weight may have benefited from the enhancement of the protein content in the feed through the addition of IPP. Consequently, the appropriate addition of IPP positively impacted the gonad growth of sea urchins.

#### 4.2.2. Effects of Adding IPP to the Feed on Gonad Color of Sea Urchins

In addition to gonad yield, gonad quality (e.g., texture, color, and taste) has become an increasingly important attribute for assessing market value [29,39,41]. Consumers tend to prefer the gonad color characterized by bright yellow, yellow–orange, and mango–orange [42]. Previous studies have shown that the brightness (L*) of sea urchin gonads fed with kelp is inferior to that of those provided with feed [12,43]. This result aligns with our findings. In the present study, the values of redness (a*) and yellowness (b*) for all feed groups were lower than those of the kelp group, resulting in increased Δ*E*_1_ and Δ*E*_2_ values for the feed groups. A lower Δ*E* value indicates a closer match to the standard color [29]. The results regarding gonad color indicated that the gonad quality of the IPP feed group remained lower than that of the kelp group. The color of sea urchin gonads is primarily linked to carotenoid intake [44,45], with kelp serving as the primary source of these compounds [46]. However, this substance demonstrated a deficiency in the feed, which may account for the inferior quality of the gonad color in all feed groups. Nevertheless, compared to the control group, the addition of IPP reduced the values of Δ*E*_1_ and Δ*E*_2_. This indicates that IPP can improve gonad color.

#### 4.2.3. Effects of Adding IPP to the Feed on Gonad Texture of Sea Urchins

In this study, the hardness, cohesiveness, gumminess, and chewiness of sea urchin gonads varied significantly among the different dietary treatments. Hardness is an important textural property, as gonads that remain intact during processing are required for high-quality products [29]. The 1.0% IPP group exhibited the highest gonad hardness. This observation may be attributed to the reduced moisture content present in their gonads. Previous studies have shown that the hardness of the gonads may be related to moisture content, as excessive moisture content causes a decrease in the hardness of *Strongylocentrotus franciscanus* [29] and *Strongylocentrotus droebachiensis* gonads [47]. We speculated that the increase in gonad hardness could be attributed to the addition of IPP, which reduced the moisture content of the gonads. At the same time, the 1.0% IPP group exhibited better cohesiveness and gumminess compared to the control group. Therefore, IPP can improve the quality of the sea urchin gonads by increasing their hardness, cohesiveness, and gumminess. In summary, IPP has the ability to address the problem of insufficient growth and quality of sea urchin gonads. However, the underlying mechanism about how IPP regulates the texture of sea urchin gonads is unknown and requires further study.

### 4.3. Effects of Adding IPP to the Feed on Antioxidant Capacity of Sea Urchins

Antioxidant enzymes play a crucial role as nonspecific immune enzymes in invertebrates, which include catalase and peroxidase [48]. Under normal physiological conditions and during instances of external stress, animals produce a variety of reactive oxygen species (ROS) within their bodies [49]. Excessive ROS may lead to oxidative stress, which is defined as a state of redox imbalance within the organism [50]. This condition can elicit inflammatory responses, thereby reducing the immune function of aquatic animals [50,51]. Following the addition of IPP, the activities of POD, CAT, and SOD demonstrated a significant increase compared to the control groups (*p* < 0.05). SOD has the ability to transform toxic oxygen ions generated by the organism into hydrogen peroxide. Subsequently, hydrogen peroxide is broken down by CAT or POD enzymes into harmless water and oxygen, thereby safeguarding the cells [52,53]. In response to physiological stress induced by microbial infections, marine organisms may enhance the expression of antioxidant enzymes to generate an immune response against pathogenic bacteria, mitigate the detrimental effects of excessive oxygen free radicals that harm the body, and sustain their normal physiological functions [54]. The enhancement of antioxidant enzyme activity undoubtedly improves the efficiency of sea urchins in converting toxic oxygen ions and their capacity to respond to microbial infections. This phenomenon may be attributed to the absorption of antimicrobial peptides in IPP by sea urchins, which has improved their immune capacity.

However, POD activity showed a declining trend as the IPP content increased from 1.0% to 1.5%. A balanced intake of nutrients is essential for the optimal functioning of biological immune functions [55]. Considering the IPP effects on the weight gain rate of sea urchins, we speculated that the high IPP content may affect their ability to absorb nutrients, thereby affecting POD activity.

### 4.4. Effects of Adding IPP to the Feed on Gut Microbiota of Sea Urchins

The gut microbiota is regarded as an organ within animals, and the intestinal microecological system is a unified entity that interacts with and relies on the surrounding environment [56,57]. The gut microbiota functions effectively only when the system achieves an optimal state of balance [58,59]. Furthermore, the gut microbiota plays an important role in animal nutrient absorption [60], defense barriers [61], and immune responses [58].

Modifications to the diet throughout the aquaculture process may result in changes to the gut microbiota of animals [5,62]. We discovered that the gut microbiota of sea urchins added with IPP is predominantly composed of *Proteobacteria* and *Campylobacterota*, which aligns with previous research [6,34]. Sea urchins fed with IPP feed exhibited an increase in the abundance of the *Firmicutes* and *Bacteroidetes* within their intestines compared to the control group. The *Firmicutes* are one of the most common types of microorganisms found in the intestines of aquatic animals [63], and they can promote the absorption of carbohydrates [64,65]. At the same time, previous studies have confirmed that *Bacteroidetes* can digest cellulose [66]. In this study, we found that the IPP group of sea urchins exhibited a significantly higher weight gain rate than the control group (*p* < 0.05). This phenomenon may be attributed to the regulation of sea urchins’ gut microbiota by IPP, which enhances the abundance of these two microorganisms and promotes their ability to absorb carbohydrates and cellulose. This may enhance the utilization of feed nutrients by the sea urchins, thereby promoting their growth.

In the 0.5% IPP group, the abundance of *Rhodobacteraceae* increased in comparison to the control group. Numerous advantageous bacteria exist in the *Rhodobacteraceae* and possess a certain degree of metabolic flexibility [67]. They not only provide energy to the host [68], but also produce antibacterial substances to prevent the incursion of pathogens and enhance the immune response of the host to a certain extent [13,69]. Among the added IPP groups, the nonspecific immunity enzymes exhibited higher levels in sea urchins compared to the control group. We speculated that this may be attributed to the increase in the abundance of *Rhodobacteraceae*.

We found that the addition of IPP reduced the abundance of *Ralstonia* in the 0.5% IPP group. *Ralstonia* is known to cause osteomyelitis and meningitis in humans. Thus, it is regarded as a potential threat to aquatic food safety [70]. It has been confirmed as a pathogen of hybrid striped bass [71], but there are currently no reports indicating whether *Ralstonia* is a potential pathogen of sea urchins. The reduction in its abundance undoubtedly exerts a positive impact on aquaculture safety and human health.

The increase in the abundance of *Vibrio* in the intestines of sea urchins after they consume feed is a concern that requires attention [13]. *Vibrio* is recognized as one of the primary pathogens of sea urchins. It has been confirmed as the causative agent of spotting disease [72,73] and black mouth disease [74]. If we can reduce their abundance, it will undoubtedly yield a positive impact on the health of sea urchins. Among all experimental groups, the abundance of *Vibrio* in the kelp group was the lowest, at merely 0.2%. Numerous carbon sources can be utilized by *Vibrio*, which tends to preferentially use these organic compounds for its proliferation, particularly when the aquaculture water environment is contaminated with organic matter [13]. Furthermore, fresh kelp contains some antibacterial substances that can reduce pathogenic outbreaks in the environment [75]. Consequently, the reduced abundance of *Vibrio* in the kelp group is likely attributable to lower feed dissolving and better water quality. In the 1.0% IPP group, the abundance of *Vibrio* reached a minimum when compared to other feed groups, but it was still higher than that of the kelp group. This phenomenon may be attributed to the absorption of antimicrobial peptides in IPP by sea urchins. We found that the addition of IPP had some positive effects on the intestinal health of sea urchins.

## 5. Conclusions

In conclusion, the addition of IPP had a positive effect on the growth, gonad quality, and antioxidant capacity. Furthermore, adding IPP to the feed can enhance gut health by decreasing the abundance of *Vibrio* and *Ralstonia*. Regarding the dosage of IPP, in order to enhance the weight gain rate, gonad quality, and intestinal health of sea urchins, we recommend adding a concentration of 1.0% IPP to the feed. Additionally, aquafarmers can add a concentration of 1.5% IPP to the feed to enhance the antioxidant capacity of sea urchins. This manuscript provides theoretical insights into the healthy farming of sea urchins.

## Figures and Tables

**Table 1:** Effect of adding IPP to the feed on survival and growth of sea urchins.

Items	Kelp Group	Feed Group			
		Control Group	0.5% IPP Group	1.0% IPP Group	1.5% IPP Group
Survival rate/%	100 ± 0	100 ± 0	100 ± 0	100 ± 0	100 ± 0
Initial body weight/g	17.26 ± 0.55	17.53 ± 0.53	17.18 ± 0.33	17.25 ± 0.58	17.14 ± 0.59
Final body weight/g	30.86 ± 0.34 ^c^	28.98 ± 0.41 ^b^	29.75 ± 0.22 ^a^	30.59 ± 0.19 ^a^	30.24 ± 0.30 ^a^
Weight gain rate/%	78.80 ± 2.11 ^c^	65.32 ± 2.14 ^b^	73.17 ± 1.82 ^a^	77.33 ± 1.73 ^a^	76.42 ± 2.16 ^a^
Wet gonad weight/g	6.20 ± 0.03 ^ab^	6.09 ± 0.06 ^b^	6.26 ± 0.02 ^ab^	6.58 ± 0.09 ^a^	6.41 ± 0.02 ^a^
Gonad index/%	20.09 ± 0.21 ^a^	21.01 ± 0.20 ^ab^	21.04 ± 0.15 ^ab^	21.51 ± 0.26 ^b^	21.20 ± 0.29 ^bc^

Note: Different lowercase letters indicate significant differences (*p* < 0.05), which apply across all findings.

**Table 2:** Effect of adding IPP to the feed on the color of sea urchin gonads.

Items	Kelp Group	Feed Group			
		Control Group	0.5% IPP Group	1.0% IPP Group	1.5% IPP Group
L* ^1^	70.69 ± 1.41 ^c^	75.99 ± 1.21 ^a^	73.12 ± 1.68 ^b^	71.72 ± 1.20 ^bc^	75.90 ± 0.99 ^a^
a* ^2^	28.12 ± 1.76 ^a^	23.09 ± 1.24 ^b^	24.78 ± 1.11 ^c^	26.51 ± 0.81 ^bc^	26.94 ± 0.66 ^bcd^
b* ^3^	45.42 ± 1.97 ^a^	40.57 ± 1.27 ^c^	41.91 ± 2.15 ^b^	44.43 ± 2.06 ^ab^	45.13 ± 0.96 ^ab^
Δ*E*_1_ ^4^	15.10 ± 0.98 ^a^	21.79 ± 1.01 ^d^	19.37 ± 0.62 ^c^	16.36 ± 0.49 ^ab^	16.89 ± 0.44 ^bc^
Δ*E*_2_ ^5^	21.05 ± 1.23 ^a^	26.18 ± 1.35 ^d^	24.55 ± 1.26 ^c^	21.97 ± 0.98 ^b^	21.08 ± 1.33 ^ab^

Notes: ^1^ L* denotes the lightness, ^2^ a* denotes the redness, ^3^ b* denotes the yellowness, ^4^ Δ*E*_1_ denotes the difference compared to the standard orange–yellow, and ^5^ Δ*E*_2_ denotes the mean color difference compared to the standard yellow. Different lowercase letters indicate significant differences (*p* < 0.05).

**Table 3:** Effect of adding IPP to the feed on the texture of sea urchin gonads.

Items	Kelp Group	Feed Group			
		Control Group	0.5% IPP Group	1.0% IPP Group	1.5% IPP Group
Hardness/N ^1^	1.30 ± 0.04 ^c^	1.21 ± 0.03 ^d^	1.31 ± 0.05 ^c^	1.41 ± 0.02 ^a^	1.35 ± 0.06 ^b^
Adhesiveness/N·mm ^2^	0.24 ± 0.01	0.26 ± 0.03	0.25 ± 0.02	0.25 ± 0.06	0.24 ± 0.03
Cohesiveness ^3^	0.23 ± 0.02 ^b^	0.23 ± 0.03 ^b^	0.24 ± 0.02 ^b^	0.27 ± 0.01 ^a^	0.26 ± 0.01 ^ab^
Springiness/mm ^4^	0.26 ± 0.02	0.24 ± 0.02	0.24 ± 0.01	0.25 ± 0.03	0.25 ± 0.01
Gumminess/N ^5^	0.30 ± 0.07 ^c^	0.27 ± 0.02 ^d^	0.31 ± 0.06 ^c^	0.37 ± 0.05 ^a^	0.35 ± 0.07 ^b^
Chewiness/mJ ^6^	0.08 ± 0.01 ^a^	0.06 ± 0.01 ^c^	0.07 ± 0.02 ^b^	0.09 ± 0.01 ^a^	0.09 ± 0.03 ^a^

Notes: ^1^ Hardness (N): the maximum peak force during the first compression of the sample. ^2^ Adhesiveness (N·mm): the work required to separate the cylindrical probe from the sample. ^3^ Cohesiveness (ratio): the internal bonding strength of the sample. ^4^ Springiness (mm): the degree to which the sample recovers after the first compression. ^5^ Gumminess (N): the viscosity characteristic of semi-solid samples (hardness × cohesiveness). ^6^ Chewiness (mJ): the work required to chew a solid sample (springiness × gumminess). Different lowercase letters indicate significant differences (*p* < 0.05).

**Table 4:** Effect of adding IPP to the feed on the routine nutritional composition of sea urchin gonads.

Items	Kelp Group	Feed Group			
		Control Group	0.5% IPP Group	1.0% IPP Group	1.5% IPP Group
GM	71.29 ± 1.21 ^c^	73.51 ± 0.98 ^a^	73.26 ± 1.48 ^ab^	70.94 ± 3.35 ^b^	71.34 ± 1.09 ^c^
CP	12.56 ± 0.44 ^d^	12.97 ± 1.12 ^cd^	13.18 ± 0.67 ^c^	13.50 ± 0.62 ^b^	13.82 ± 0.92 ^a^
CF	5.06 ± 0.51 ^a^	4.80 ± 0.66 ^b^	4.84 ± 0.78 ^b^	5.01 ± 0.74 ^a^	4.96 ± 1.01 ^ab^

Note: “GM” means gonad moisture, “CP” means crude protein, and “CF” means crude fat. Different lowercase letters indicate significant differences (*p* < 0.05).

**Table 5:** Effect of adding IPP to the feed on the *α*-diversity of sea urchin gut microbiota.

Items	KELP Group	Feed Group			
		Control Group	0.5% IPP Group	1.0% IPP Group	1.5% IPP Group
ACE	203.09 ± 31.73 ^e^	672.87 ± 87.17 ^b^	872.2 ± 104.34 ^a^	479.84 ± 65.7 ^c^	283.12 ± 42.15 ^d^
Chao1	201.2± 62.29 ^e^	678.06 ± 75.19 ^b^	850.80 ± 96.1 ^a^	481.77 ± 67.9 ^c^	289.11 ± 60.07 ^d^
Shannon	2.16 ± 0.36 ^c^	2.11 ± 0.32 ^c^	2.69 ± 0.38 ^ab^	3.45 ± 0.42 ^a^	2.61 ± 0.38 ^ab^
Simpson	0.25 ± 0.04 ^a^	0.29 ± 0.05 ^a^	0.21 ± 0.04 ^b^	0.11 ± 0.04 ^c^	0.15 ± 0.03 ^c^

Note: The *α*-diversity index of the gut microbiota in sea urchins fed experimental feed for 60 days: “ACE” means “abundance-based coverage estimator”. Different lowercase letters indicate significant differences (*p* < 0.05).

## Data Availability

The original contributions presented in the study are included in the article. If there is a request, further inquiries can be directed to the corresponding author.

## Supplementary Materials

The following supporting information can be downloaded at: https://www.mdpi.com/article/10.3390/biology14070874/s1, Table S1. Formulation and proximate composition of the experimental diets (% dry matter); Table S2. The definition of texture evaluation indicators.
