# Supplementary material for: Effects of Dietary Tussah Immunoreactive Pupa Powder on Growth, Gonad Quality, Antioxidant Capacity, and Gut Microbiota of the Sea Urchin Strongylocentrotus intermedius
Source: Biology (Basel). 2025 Jul 17;14(7):874. doi: 10.3390/biology14070874 (PMC12292499; doi:10.3390/biology14070874)
Supplement: Supplementary file 1 [file biology-14-00874-s001.zip › Table S1. Formulation and proximate composition of the experimental diets (% dry matter)..pdf]

Table S1. Formulation and proximate composition of the experimental diets (% dry matter).

Items	Feed group			
	Control group	0.5% IPP group	1.0% IPP group	1.5% IPP group
Fish meal [1]	4.00	4.00	4.00	4.00
Soybean meal [2]	13.00	13.00	13.00	13.00
Wheat meal [3]	24.00	24.00	24.00	24.00
Wheat bran [4]	20.00	20.00	20.00	20.00
Corn starch [5]	20.70	20.20	19.70	19.20
Wheat gluten [6]	10.00	10.00	10.00	10.00
Vitamin premix [7]	2.00	2.00	2.00	2.00
Mineral premix [8]	2.00	2.00	2.00	2.00
IPP	0.00	0.50	1.00	1.50
Calcium propionate	0.18	0.18	0.18	0.18
Ethoxyquin	0.02	0.02	0.02	0.02
Choline chloride	0.10	0.10	0.10	0.10
Palmoil	4.00	4.00	4.00	4.00
Proximate composition	-	-	-	-
Crude protein	26.81	26.84	26.90	26.92
Crude lipid	6.51	6.56	6.59	6.61

Note: [1] Fish meal: Crude protein 66.3%, crude lipid 9.4%; [2] Soybean meal: Crude protein 51.6%, crude fat 0.9%. [3] Wheat flour: Crude protein 13.66%. [4] Wheat bran: Crude protein 19.2%, crude fat 4%. [5] Corn starch: Crude protein 20.4%, crude fat 5%. [6] Vital wheat gluten: Crude protein 68.7%, crude fat 2.8%. [7] Vitamin premix (mg or g/kg diet): Vitamin D, 5 mg; Vitamin K, 10 mg; Vitamin B12, 10 mg; Vitamin B6, 20 mg; Folic acid, 20 mg; Vitamin B_1, 25 mg; Vitamin A, 32 mg; Vitamin B_2, 45 mg; Pantothenic acid, 60 mg; Biotin, 60 mg; Niacin, 200 mg; Vitamin E, 240 mg; Inositol, 800 mg; Vitamin C, 2000 mg; Microcrystalline cellulose, 16.47 g. [8] Mineral premix (mg or g/kg diet): $CuSO_4 \cdot 5H_2O$, 10 mg; $ZnSO_4 \cdot H_2O$, 50 mg; $FeSO_4 \cdot H_2O$, 80 mg; $MnSO_4 \cdot H_2O$, 45 mg; $CoCl_2 \cdot 6H_2O$ (1%), 50 mg; $NaSeSO_3 \cdot 5H2O$ (1%), 20 mg; $Ca(IO_3)_2 \cdot 6H_2O$ (1%), 60 mg.
