# Supplementary material for: Effects of Dietary Tussah Immunoreactive Pupa Powder on Growth, Gonad Quality, Antioxidant Capacity, and Gut Microbiota of the Sea Urchin Strongylocentrotus intermedius
Source: Biology (Basel). 2025 Jul 17;14(7):874. doi: 10.3390/biology14070874 (PMC12292499; doi:10.3390/biology14070874)
Supplement: Supplementary file 1 [file biology-14-00874-s001.zip › Table S2. The definition of texture evaluation indicators.pdf]

Table S2. The definition of texture evaluation indicators

Items	Define
Hardness (*N*)	The maximum peak force was observed during the initial compression of the sample
Adhesiveness (*N.mm*)	The work needed to separate the cylindrical probe from the sample
Springiness (*mm*)	The degree to which the sample recovered after the initial compression
Cohesiveness (*ratio*)	The internal bonding strength of the sample
Cohesiveness (*ratio*)	The viscosity characteristic of semi-solid samples (Hardness × Cohesiveness)
Chewiness (*mJ*)	The work needed to chew a solid sample (Springiness × Gumminess)
